# Comparative evaluation of iodinated, nanoparticle-based, and gadolinium-based contrast agents in computed tomography for small animals

**DOI:** 10.1371/journal.pone.0350386

**Published:** 2026-06-29

**Authors:** Julie van Krimpen Mortensen, Emil Svendborg Christensen, Amalie Littau, Nicoline Rosenkilde Kjaer, Sophie Arndt, Anne Skovsbo Clausen, Andreas Clemmensen, Andreas Kjaer

**Affiliations:** Department of Clinical Physiology and Nuclear Medicine & Cluster for Molecular Imaging, Copenhagen University Hospital – Rigshospitalet & Department of Biomedical Sciences, University of Copenhagen, Denmark; Ural Federal University named after the first President of Russia B N Yeltsin Institute of Physics and Technology: Ural’skij federal’nyj universitet imeni pervogo Prezidenta Rossii B N El’cina Fiziko-tehnologiceskij institut, RUSSIAN FEDERATION

## Abstract

Preclinical computed tomography (CT) is extensively used for anatomical reference and multimodal imaging, but the soft tissue contrast is poor compared to clinical CT. This hinders reliable organ delineation and segmentation in small-animal studies. We present a systematic evaluation of commonly used clinical iodinated and gadolinium-based contrast agents, together with a preclinical nanoparticle-based contrast agent. This evaluation aims to enhance soft-tissue boundary definition and in vivo visibility. The study consisted of two parts. First, phantom experiments were performed to assess the relationship between contrast-agent concentration and Hounsfield Unit (HU) across multiple dilutions and CT energy settings. Second, these findings were evaluated in vivo in mice to assess organ-level enhancement. Phantom measurements demonstrated a linear relationship between HU and concentration for all agents; however, at higher dilutions, contrast enhancement became indistinguishable from the background. In vivo, clinical iodinated and gadolinium-based agents administered intraperitoneal (IP) provided enhanced soft-tissue contrast by outlining abdominal organs, whereas the intravenously administered nanoparticle agent exhibited pronounced organ-specific accumulation. Overall, IP injection of clinical iodinated contrast agents offers a cost-effective and practical strategy for enhancing organ boundary visibility in preclinical CT. For longitudinal or organ-specific imaging studies, the preclinical nanoparticle-based contrast agent remains advantageous, as its cumulative uptake allows detection over extended periods and minimizes burden from multiple injections.

## Introduction

Preclinical imaging is essential for bridging the gap between laboratory research and clinical practice. This approach advances the development of novel diagnostic and therapeutic tools for clinical applications, and these studies are predominantly conducted in rodents, often using systems such as Positron Emission Tomography and Computed Tomography (PET/CT). Quantifying images relies on manually segmenting relevant organs from the CT image [1]. The quantification of CT images is based on the Hounsfield Unit (HU) [2], and differentiating soft tissue structures in these images can be particularly challenging due to their similar HU values [3]. As a result, manual segmentation becomes a laborious process, prone to inaccuracies and variability between annotators.

The primary option for enhancing soft tissue contrast is the use of contrast agents, which are categorized based on their chemical structure. Contrast agents used in clinical practice are most often iodine-based [4]. Due to the higher Z-number (Z = 53) of iodine, attenuation will increase in areas where it is present, thereby increasing contrast through increased HU value [5]. Iodinated contrast agents have been used in CT imaging for many years, and their use has been broadly characterized in the literature, particularly concerning their physicochemical properties, safety considerations, and diagnostic performance, which is discussed in detail elsewhere [4,6–8].

Gadolinium (Z = 64) is commonly used as a contrast agent in magnetic resonance imaging (MRI) due to its magnetic properties [4,5]. Having a relatively high Z-number, we hypothesized that the use of gadolinium could increase soft tissue contrast for CT imaging.

Contrast agents are often administered intravenously (IV) to ensure fast systemic circulation [3], which requires establishing venous access. Furthermore, contrast agents specifically developed for preclinical studies are often expensive, while those developed for clinical use, though more easily available and affordable, are rapidly metabolized in rodents, significantly shortening the available imaging window.

Intraperitoneal (IP) administration provides a convenient alternative to IV administration in small-animal CT imaging. IP injection can result in temporary pooling of contrast agent within the peritoneal cavity, outlining abdominal organs and their boundaries prior to systemic absorption. Additionally, in PET/CT workflows, where IV access is required for radiotracer delivery and injectable volumes are constrained in small animals, IP injection further enables anatomical enhancement without exceeding IV volume limits. The ability of iodinated contrast agents to cross the peritoneal membrane and enter the bloodstream is influenced by their physicochemical properties, particularly molecular weight, osmolarity, and water solubility. Low-molecular-weight and water-soluble agents, such as non-ionic monomeric compounds, tend to exhibit more favorable pharmacokinetics for systemic absorption, although this process is also dependent on membrane integrity [8].

In this study, we systematically compare contrast agents designed for clinical and preclinical CT imaging in mice, and propose methods to use IP injection of clinical iodinated contrast agents to enhance soft-tissue boundaries. Our goal is to offer a widely applicable disease-agnostic method for improving anatomical contrast to support organ segmentation in varied preclinical workflows. By obtaining better soft-tissue contrast, this approach can enhance manual segmentation, both reducing the time required and improving the quality of the resulting annotations. The experimental overview and in vivo example results are presented in S1 Fig

## Materials and methods

### Contrast agents

Five different contrast agents were tested: Iomeron (S.p.A. Bracco, Milan, Italy), Omnipaque (GE Healthcare AS, Chicago, Illinois, USA), Visipaque (GE Healthcare AS, Chicago, Illinois, USA), Gadovist (Bayer, Copenhagen, Denmark), and ExiTron nano 12000 (Viscover, Berlin, Germany). A summary of the primary properties of the different contrast agents is presented in S1 Table.

### Phantom study

Stock solutions of the contrast agents were diluted in Milli-Q ultrapure water to generate a series of concentrations with the following dilution ratios: 1:0, 1:1, 1:3, 1:4, 1:9, 1:19, 1:24, 1:49, 1:99, 1:199. Each dilution was transferred into a 0.5 mL safe lock microtube (Eppendorf, Hamburg, Germany), which served as the imaging phantoms. The phantoms were placed in a custom-designed, 3D-printed holder, fabricated using a Prusa MK3s 3D printer and PETG filament (Prusa Research, Prague, Czech Republic).

All phantoms were CT-scanned on an Inveon PET/CT scanner (Siemens Healthineers, Knoxville, USA) using two main protocols: 50 kV and 880 ms exposure time, along with 80 kV and 270 ms exposure time. Both protocols used 500 μA current, 4x4 binning, and 360 projections. All images were reconstructed using the vendor-provided Feldkamp algorithm.

The CT images were analyzed by drawing regions of interest (ROIs) of identical size in each phantom and extracting the mean HU values using Python (version 3.9, Beaverton, OR, USA). All data are presented as mean ± standard deviation (SD), and illustrations created using Prism version 10.5.0 (GraphPad software, Boston, Massachusetts, USA).

### Animal study

Six-week-old female Rj:NMRI-Foxn1^nu/nu^ were purchased (Janvier, Le Genest-Saint-Isle, France) and housed in groups of 4–8 mice in individually ventilated cages under regular lighting conditions. The animals received a standard pathogen-free pellet diet and water ad libitum. All animal experiments were performed according to Directive 2010/63/EU of the European Parliament and the European Council on the protection of animals used for scientific purposes. The Animal Experiments Inspectorate in Denmark approved the experiments under an approved animal license (2021–15-0201-01041, approved December 15, 2021), as well as the institutional animal welfare body. All animal experiments were conducted and reported according to PREPARE and ARRIVE guidelines [9,10]. Animals were monitored weekly for signs of distress, including excessive weight loss (>20%) or signs consistent with predefined humane endpoints. At study termination, animals were euthanized by cervical dislocation. All efforts were made to minimize discomfort and ensure animal welfare throughout the experiment.

### CT imaging of animals

**Fig 1:**
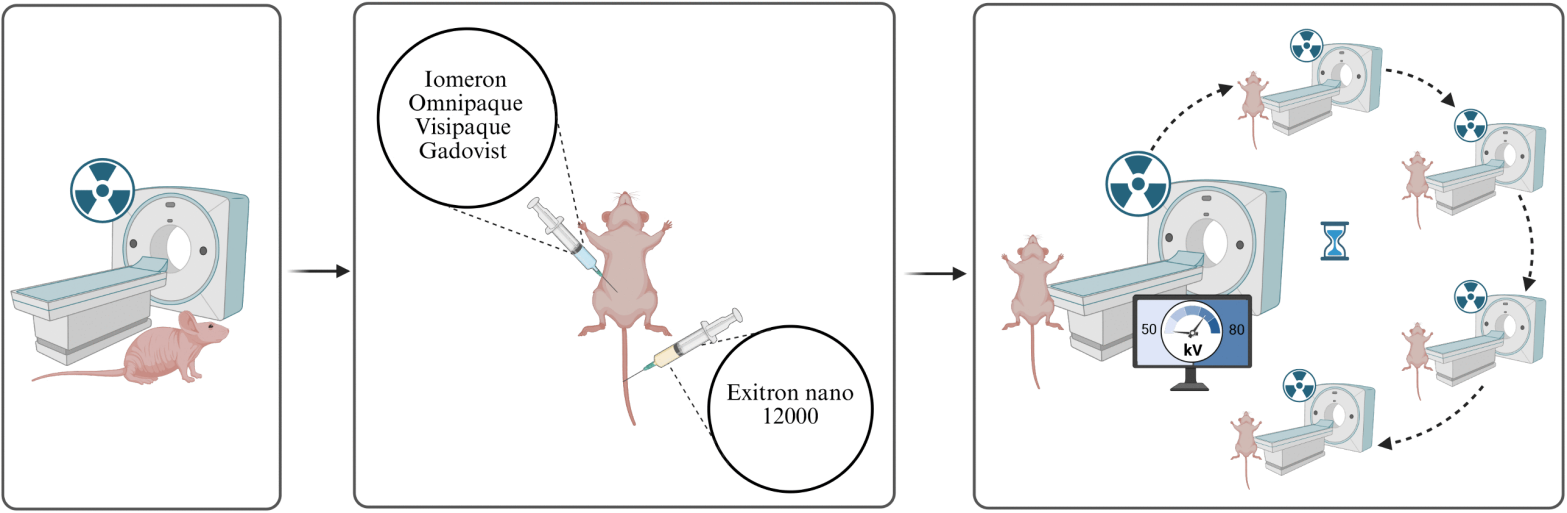
Overview of the in vivo study. Animals were first imaged with CT without contrast enhancement. Subsequently, the animals received intraperitoneal injections of either Iomeron, Omnipaque, Visipaque, or Gadovist, or an intravenous injection of ExiTron nano 12000, at varying dilutions. Immediately following the injections, animals were subjected to five consecutive CT scans using either the 50 kV or 80 kV protocol.

Animals were anesthetized with 3–4% sevoflurane in a gas mixture of 65% nitrogen (N_2_) and 35% oxygen (O_2_) and positioned in a four-animal bed system. 200 μL solution, consisting of Iomeron, Visipaque, Omnipaque, and Gadovist in varying dilutions (1:0, 1:4, 1:9, 1:19), was administered as an IP injection. Immediately following injection, five consecutive CT acquisitions were performed using either the 50 kV or 80 kV protocol. The duration of the two protocols was 11 min for the 50 kV protocol and 7 min for the 80 kV protocol. Each protocol was conducted for five consecutive repetitions, resulting in a total scan time of 55 min and 35 min, respectively. ExiTron nano 12000 was administered IV via the tail vein at a dose of 100 μL, using the same dilution series and imaging protocol as for the IP injections. An overview of the in vivo study is depicted in Fig 1.

ROIs were manually delineated on the reconstructed CT images for the liver, muscle, kidney, stomach, spleen, and blood. The blood ROI was placed within the left ventricle of the heart to obtain a representative measure of intravascular contrast. Mean HU values were extracted for each ROI across all contrast dilutions and time points.

The relationship between contrast agent concentration and HU was analyzed across all different dilutions, as well as the temporal changes in HU across the five consecutive CT protocols.

To account for image-specific noise variations and to enable comparison across scans, the mean HU measurements were normalized to background noise. Two rectangular background ROIs were placed in homogeneous regions at the top and bottom of each CT scan, and the SDs were extracted. The organ ROI values were normalized by dividing the mean HU of the organ by the SD of the background. Normalized values were evaluated at the third scan time for each protocol (approximately 33 min for the 50 kV protocol and 21 min for the 80 kV protocol). A minimum effective dose (MED) threshold was defined at 2 and was used to indicate the minimum contrast level at which an effect is observed.

## Results

### Phantom study

**Fig 2:**
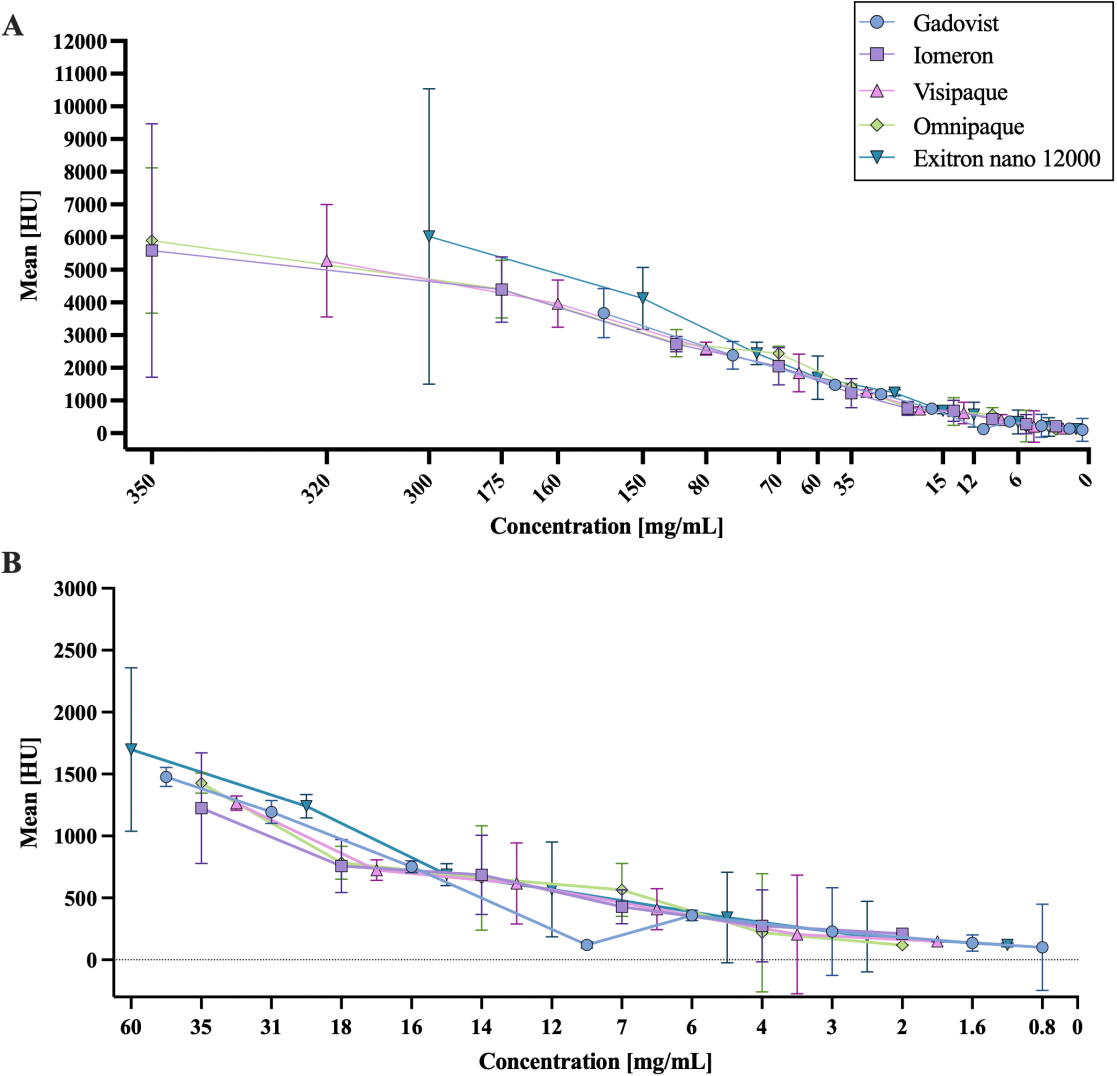
Change in mean CT contrast in phantoms. **(A)** Hounsfield Units (HU) for the different concentrations of contrast agents using the 50 kV scan protocol. **(B)** The tail of the curve, zoomed in such that the data starts from 60 mg/mL. Presented as mean values and standard deviation.

The results from the phantom study using the 50 kV protocol are presented in Fig 2, which illustrates how the concentration of the contrast agent affects the mean HU values for the five different contrast agents. Fig 2A displays the changes from highest to lowest concentration (350 − 0 mg/mL), while Fig 2B focuses on the range from 60 to 0 mg/mL to highlight subtle variations at lower concentrations. The contrast agent with the highest HU value is ExiTron nano 12000, which, at a concentration of 300 mg/mL, has a HU value of 6,500. The second-highest of all the contrast agents is the iodine-based Omnipaque, which at 350 mg/mL has a HU value of 6,000. This is followed by Iomeron, Visipaque, and Gadovist. The equivalent results from the 80 kV protocol are presented in the supplementary material S2 Fig.

### Animal study

**Fig 3:**
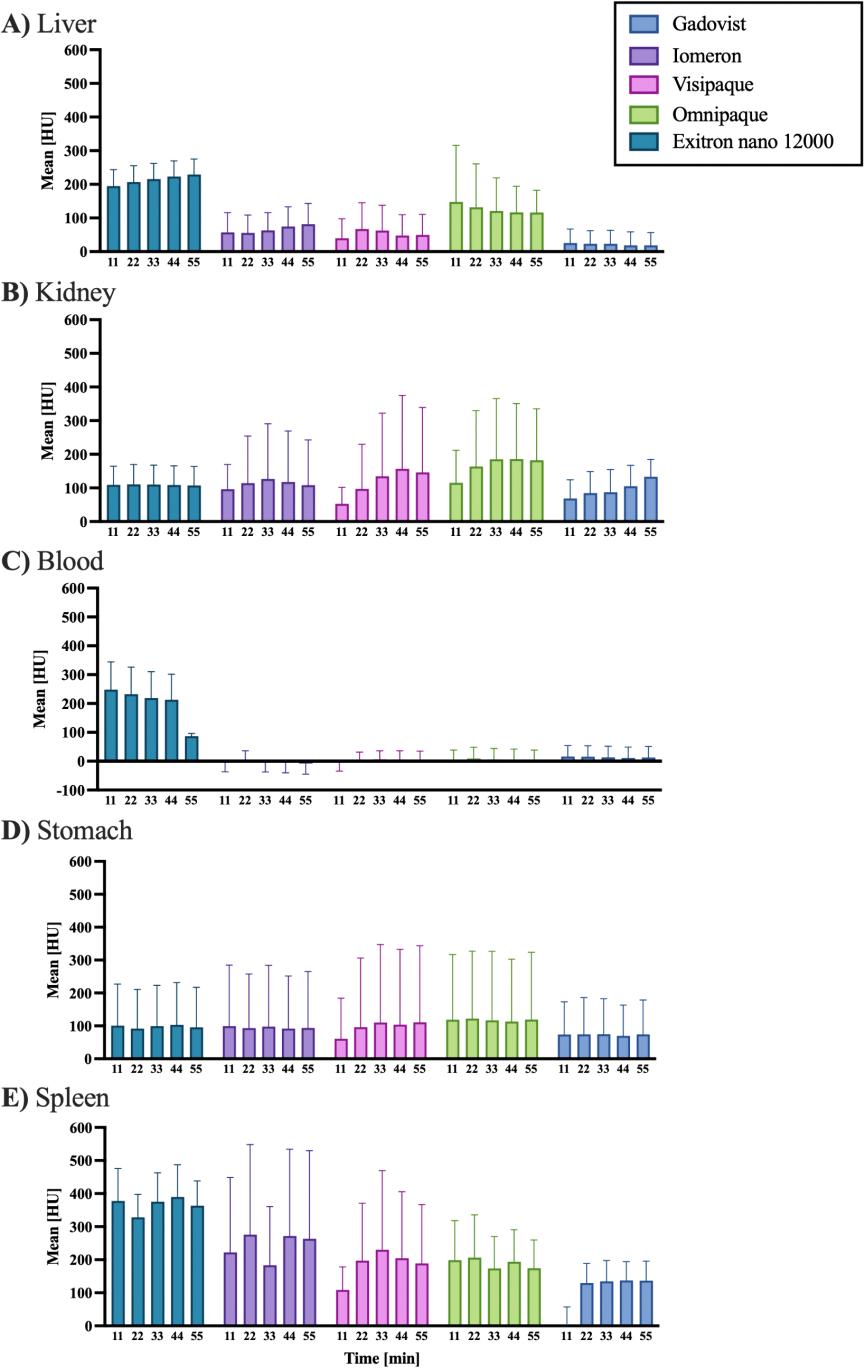
Mean HU values across five consecutive CT scans for five contrast agents. Mean HU values, using the 50 kV protocol, are shown for each contrast agent in three regions of interest: (A) liver, (B) kidney, (C) blood, (D) stomach, and (E) spleen.

Fig 3 illustrates the mean HU values across five consecutive CT scans for the five contrast agents using the 50 kV protocol in vivo, showing the HU value of the liver, kidney, blood, stomach, and spleen in parts A, B, C, D, and E, respectively. The liver (Fig 3A), blood (Fig 3C), and spleen (Fig 3E) show the highest HU value when using ExiTron nano 12000 compared to the other contrast agents at all timepoints. All contrast agents except ExiTron nano 12000 show an increased concentration in the kidneys over time, whereas Iomeron and Visipaque also show a decrease after a peak in intensity around 33–44 minutes. The HU value of the blood is constant at around 0 HU for all contrast agents except ExiTron nano 12000, where a decrease from 250 to around 100 HU is observed.

**Fig 4:**
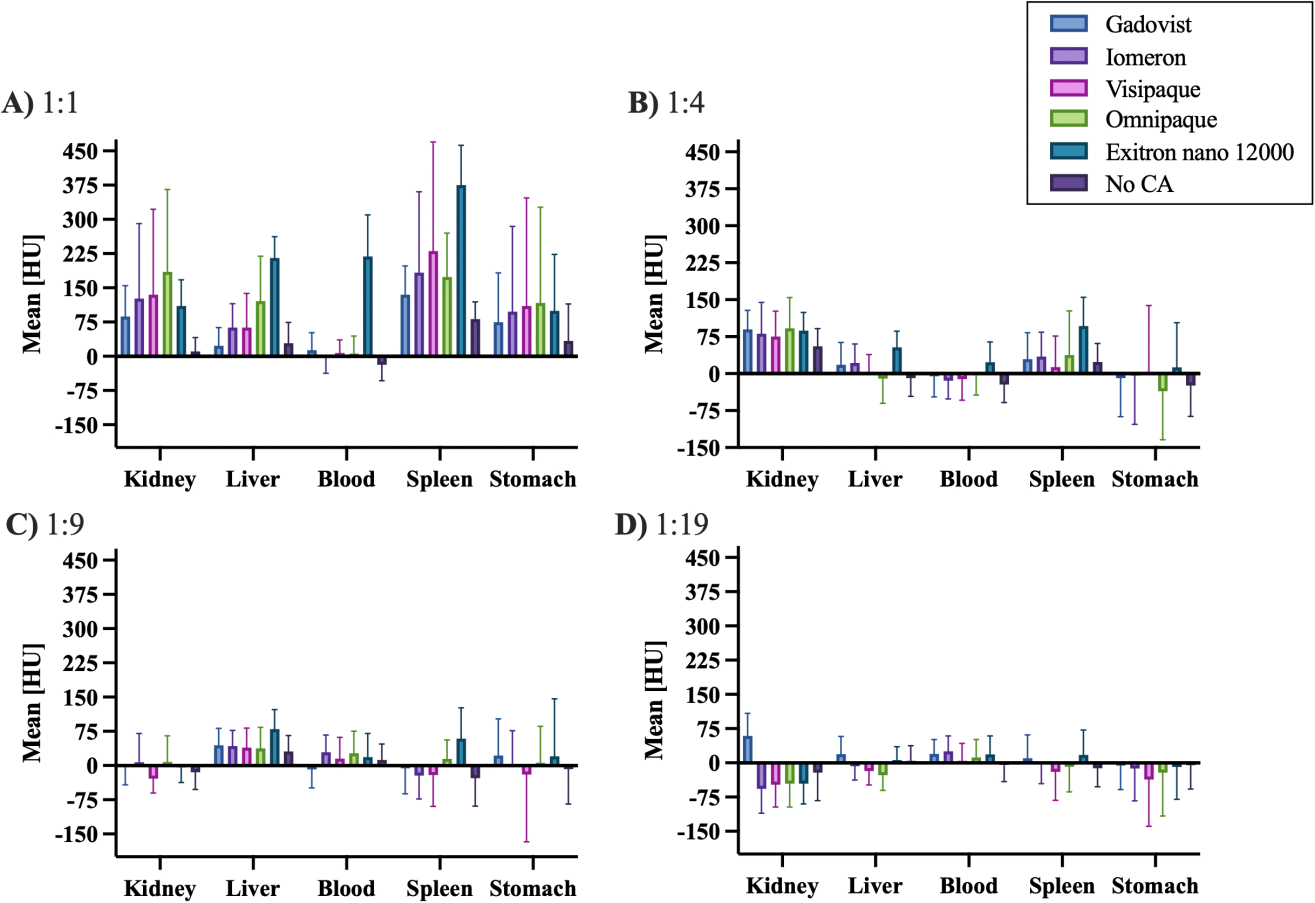
Mean HU values in the kidney, liver, blood, stomach, and spleen. Values at 33 minutes post-injection of the five different contrast agents obtained using the 50 kV protocol. (A) 1:0 dilution ratio, (B) 1:4 dilution ratio, (C) 1:9 dilution ratio, and (D) 1:19 dilution ratio.

An overview of contrast agent dilutions and mean HU values at 33 minutes post-injection are shown in Fig 4. The data include measurements from the liver, kidney, blood, stomach, and spleen following administration of the contrast agent at four different dilutions: 1:0, 1:4, 1:9, and 1:19 in parts (A), (B), (C), and (D), respectively. A notable difference in HU values between the non-diluted (Fig 4A) and diluted contrast agents (Fig 4B-D) is observed.

**Fig 5:**
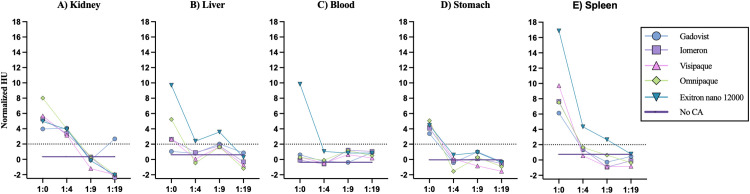
Normalized HU values in the kidney, liver, blood, stomach, and spleen. Values at 33 minutes post-injection of the five different contrast agents at dilutions 1:0, 1:4, 1:9, and 1:19, obtained using the 50 kV protocol. Normalized to background standard deviation. Each panel (A–E) represents a different organ. The horizontal dashed line at y = 2 indicates the minimum effective dose threshold.

Normalized mean HU values for the five contrast‑agent dilutions at 33 minutes post‑injection are shown in Fig 5 for each organ. A horizontal dashed line at y = 2 marks the MED threshold. Depending on the organ of interest and contrast agent, a dilution of 1:4 is shown to be below the threshold of 2. Only the kidneys (Fig 5A) show values above the threshold at a dilution of 1:4.

**Fig 6:**
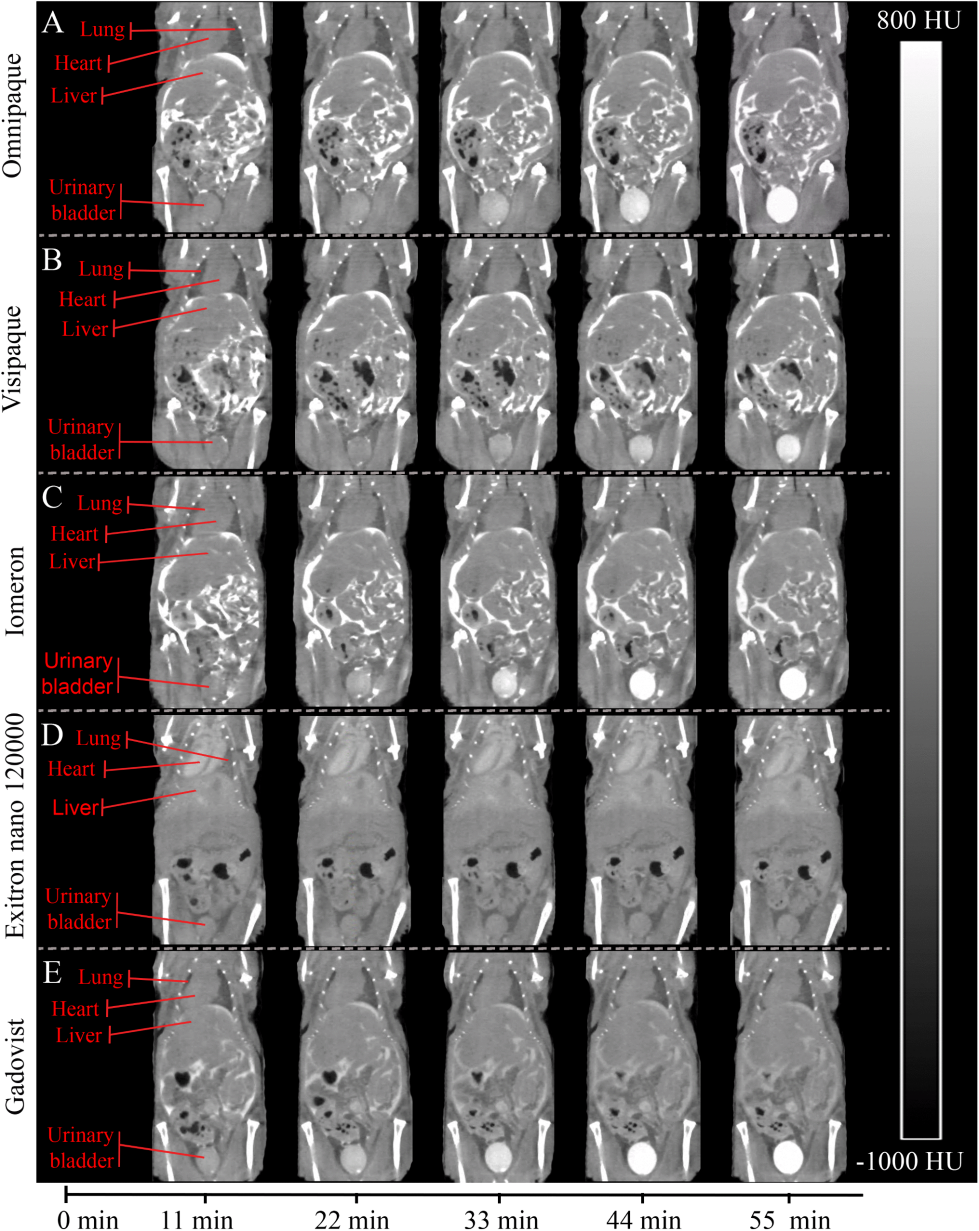
Representative CT images acquired using the 50 kV protocol. Images show mice administered with each of the five different undiluted contrast agents at various time points post-injection.

Representative CT images of mice injected with undiluted contrast agents are shown in Fig 6. Each row corresponds to a different agent, illustrating the distribution and enhancement patterns over time. For Omnipaque, Visipaque, Iomeron, and Gadovist, progressive contrast accumulation in the bladder is observed, as indicated by an increase in signal intensity over time. Additionally, Omnipaque, Visipaque, and Iomeron exhibit pronounced contrast enhancement in the peritoneal region, particularly surrounding the liver and intestines, indicated by the bright signal in these regions. In contrast, Gadovist shows comparatively lower peritoneal enhancement, with the signal predominantly localized to the bladder. Conversely, ExiTron nano 12000 demonstrates a distinct contrast enhancement pattern, with the signal primarily localized within the organs. Notably, the liver appears more clearly delineated, and the heart is more distinctly visualized compared to the other agents. Results obtained using the 80 kV imaging protocol are presented in the supplementary material (S3 Fig-S6 Fig).

## Discussion

The presented work compares commonly available contrast agents for preclinical CT imaging and provides a proof of concept for IP administration of clinical iodinated agents in mice. IP-injected contrast agents improved soft-tissue visibility by outlining abdominal organ boundaries, whereas the nanoparticle-based ExiTron nano 12000, designed for IV use, produced organ-specific contrast enhancement. This qualitative effect was evaluated using both phantom and in vivo experiments.

The purpose of this study is to improve organ visibility, either by outlining the organ boundary or by increasing the organ-specific contrast, rather than to detect lesions. Lesion-specific enhancement requires different pharmacokinetic properties and therefore falls outside the general scope of our methodological aim.

### Linearity of contrast agents dilution in phantoms

A linear relationship between the concentration of contrast agents and HU values was demonstrated in phantoms, supporting the feasibility of diluting contrast agents while maintaining predictable imaging outcomes. However, below a certain concentration, the quantitative effect becomes negligible. As shown in Fig 2, the attenuation curve converges at around 70 mg iodine/mL, making the agents difficult to distinguish from one another.

### Dilution strategies and cost efficiency

In the in vivo experiment, the strongest effects were observed with undiluted contrast agents, as shown in Fig. 4A. By normalizing the HU data (Fig. 5), we enabled direct organ-to-organ comparisons while accounting for inherent differences among contrast agents and CT imaging protocols. This normalization allowed for a quantitative definition of a MED. A MED threshold of 2 was visually selected as the minimum level at which the contrast agent effect remained detectable. For reference, a commonly accepted threshold for robust visual detectability is the Rose criterion of 5, which represents a clearly discernible contrast difference [11]. In contrast to studies focused on maximum detectability, our objective was to identify conditions under which contrast enhancement becomes minimally detectable or indistinguishable.

We observed that, depending on the organ and contrast agent, substantial dilution could be applied without compromising segmentation performance. For example, a dilution of 1:4 was sufficient for spleen segmentation when using ExiTron nano 12000. This is particularly relevant given that ExiTron nano 12000 is the most expensive contrast agent evaluated in this study.

Dilution of contrast agents for in vivo imaging can significantly reduce costs, especially in high-throughput or longitudinal studies. This consideration is most relevant for ExiTron nano 12000, whereas Omnipaque, Iomeron, and Visipaque are relatively inexpensive and generally do not require dilution for cost-efficiency. Nevertheless, dilution introduces a trade-off between reduced cost and image quality, as excessive dilution may compromise contrast enhancement, as demonstrated in Figs. 4 and 5. Importantly, dilution also lowers the iodine burden per animal, which may be beneficial from both toxicity and ethical perspectives.

Although Gadovist is not designed as a CT contrast agent, it produced modest soft-tissue contrast when administered undiluted. However, it was consistently outperformed by all iodine-based agents and is comparatively expensive (S1 Table). Consequently, Gadovist is not recommended for segmentation-oriented CT imaging.

### Influence of administration routes

The choice of administration route strongly affects the distribution of the contrast agent and its organ-specific uptake. ExiTron nano 12000 was administered IV, while all other contrast agents were given IP. Due to differences in formulation and administration route, direct comparisons should be interpreted with caution.

IP delivery of iodinated clinical agents was chosen because these typically exhibit rapid systemic washout when given IV in mice, providing a short imaging window. In contrast, IP injection results in peritoneal pooling that outlines the abdominal organs and, depending on molecular properties, may lead to delayed systemic absorption. In addition, differences in formulation osmolarity may induce transient osmotic water shifts following IP administration, thereby influencing early peritoneal dilution and contrast distribution. This pooling around the abdominal organs at the site of initial localization can be observed in Fig 6. Quantitatively, absorption of the IP injected agents resulted in increasing HU values in absorbing organs (Fig 4).

While IP injections are fast, minimally invasive, and practical, their effectiveness depends on the physicochemical properties of the agent and the specific aims of the imaging study. Highly hyperosmolar agents may promote greater peritoneal fluid recruitment, whereas iso‑osmolar formulations are expected to exert minimal osmotic effects, potentially contributing to differences in early imaging behavior. Formulations ranged from iso‑osmolar (Visipaque) to markedly hyperosmolar (Gadovist) relative to plasma (290 mOsm/kg) [12] (S1 Table). Although IP administration can be advantageous for imaging the peritoneal space, it provides less predictable systemic distribution than IV administration and is therefore not suitable for applications requiring precise pharmacokinetic timing.

In molecular imaging workflows, IV access is required for radiotracer delivery, and the total injectable volume is a critical constraint in small animals. Under these constraints, IP injection of contrast provides a practical strategy for enhancing soft‑tissue visibility without exceeding IV volume limits or the number of IV procedures.

From a biological perspective, few injections at the same site are generally well tolerated, but as with any administration route, repeated dosing may increase the risk of local irritation. For studies requiring repeated or long-term contrast enhancement, it is recommended to use alternative strategies.

### Application-specific use of contrast agents

The choice of contrast agent depends on the experimental objective, including the target organ, study duration, and whether repeated imaging is required. ExiTron nano 12000 provides strong and prolonged liver and spleen enhancement after a single IV injection [13,14], making it especially suitable for longitudinal studies where repeated dosing is undesirable.

The study was intentionally designed to be disease‑agnostic to maintain broad applicability across research settings. Introducing a single disease model would narrow the method’s applicability and require additional disease-specific comparisons. Instead, the generalized strategy presented here is intended to be applicable across different disease models and research laboratories.

### Temporal resolution and imaging windows

Five consecutive CT scans were used to capture the early contrast dynamics. This design captured the early phase of contrast distribution, but may have missed delayed peaks, especially for ExiTron nano 12000, which is known to exhibit prolonged retention [4,15]. This behavior suggests that peak contrast enhancement may occur several hours post-injection, and extended imaging windows would therefore better characterize the late-phase enhancement.

Due to the difference in temporal resolution of the CT protocols, a specific imaging window for peak contrast cannot be given. We recommend a renal imaging window of approximately 30 minutes following IP administration of iodinated agents.

### Impact on image segmentation

Contrast enhancement can improve the definition of organ boundaries. While this study does not include an Artificial intelligence (AI) segmentation pipeline, the demonstrated enhancement from IP contrast can facilitate more accurate manual segmentation and may support future AI development. AI-based segmentation has great potential for addressing the challenges of manual segmentation and is increasingly being utilized in preclinical research [1,16]. However, effective application requires training on large datasets that also capture a wide range of variability to reflect the diversity of varying imaging conditions.

## Conclusion

This study presents a comparative evaluation of various contrast agents for small-animal CT imaging, aiming to establish a generalizable disease-agnostic method for improving organ delineation across preclinical applications.

Contrast‑agent dilution showed linear attenuation behavior in phantoms. In vivo, dilution reduces cost and iodine burden, although the strongest enhancement was consistently achieved with undiluted agents. The MED threshold of 2 showed that the clinical contrast agents should not be diluted in most cases. Primarily, Exitron nano 12000 could be diluted 1:4 when interested in the spleen.

Clinical iodinated contrast agents, such as Omnipaque, Iomeron, and Visipaque, provide reasonable contrast enhancement following IP injection, with signal primarily in the peritoneal cavity surrounding the abdominal organs. The agents are widely available, inexpensive options suitable for routine imaging and multimodal imaging workflows. In contrast, the nanoparticle-based ExiTron nano 12000 demonstrated superior organ-specific enhancement, particularly in the liver, heart, and spleen. Gadovist is not recommended for CT imaging due to inferior performance and higher costs relative to the iodinated contrast agents.

Overall, the choice of contrast agent should be guided by study design, imaging goals, and available resources.

## Supporting information

S1 Table Different properties of the five contrast agents. Includes structural form, ionicity, standard concentration, molecular weight, osmolarity, and viscosity at different temperatures. N/A indicates that the information is not available or not disclosed by the vendor.
(PDF)

S1 Fig Striking image. Overview of the experimental setup and representative contrast enhanced CT images of the included contrast agents.
(TIF)

S2 Fig Change in mean CT contrast in phantoms. (A) Hounsfield Units (HU) for the different concentrations of contrast agents using the 80 kV scan protocol. (B) The tail of the curve, zoomed in such that the data starts from 60 mg/mL. Presented as mean values and standard deviation.
(TIF)

S3 Fig Mean HU values across five consecutive CT scans for five contrast agents. Mean HU values, using the 80 kV protocol, are shown for each contrast agent in three regions of interest: (A) liver, (B) kidney, (C) blood, (D) stomach, and (E) spleen.
(TIF)

S4 Fig Mean HU values in the kidney, liver, blood, stomach, and spleen. Values at 21 minutes post-injection of the five different contrast agents obtained using the 80 kV protocol. (A) 1:0 dilution ratio, (B) 1:4 dilution ratio, (C) 1:9 dilution ratio, and (D) 1:19 dilution ratio.
(TIF)

S5 Fig Normalized HU values in the kidney, liver, blood, stomach, and spleen. Values at 21 minutes post-injection of the five different contrast agents at dilutions 1:0, 1:4, 1:9, and 1:19, obtained using the 80 kV protocol. Normalized to background standard deviation. Each panel (A–E) represents a different organ. The horizontal dashed line at y = 2 indicates the minimum effective dose threshold.
(TIF)

S6 Fig Representative CT images acquired using the 80 kV protocol. Images show mice administered each of the five different contrast agents at various time points post-injection.
(TIF)
