# Supplementary material for: Comparative evaluation of iodinated, nanoparticle-based, and gadolinium-based contrast agents in computed tomography for small animals
Source: PLoS One. 2026 Jun 29;21(6):e0350386. doi: 10.1371/journal.pone.0350386 (PMC13313363; doi:10.1371/journal.pone.0350386)
Supplement: S1 Table — Includes structural form, ionicity, standard concentration, molecular weight, osmolarity, and viscosity at different temperatures. N/A indicates that the information is not available or not disclosed by the vendor. (PDF) [file pone.0350386.s001.pdf]

	**Iomeron**	**Omnipaque**	**Visipaque**	**Gadovist**	**ExiTron nano 12000**
**Structural form**	Monomeric	Monomeric	Dimeric	Macrocyclic chelate	Nanoparticle
**Ionicity**	Non-ionic	Non-ionic	Non-ionic	Non-ionic	Non-ionic
**Concentration**	350 mgI/mL	350 mgI/mL	320 mgI/mL	157.25 mgGd/mL	N/A
**Molecular weight (15)**	732.64	884.00	1550.00	604.71	N/A
**Osmolarity [mOsm/kg]**	618.00	780.00	290.00	1603.00	N/A
**Viscosity at 20 °C [mPa·s]**	14.50	20.40	26.60	8.86	N/A
**Viscosity at 37 °C [mPa·s]**	7.50	10.40	11.80	4.96	N/A
**Price [€/mL]**	0.64	0.37	0.46	8.75	898.23
