# Supplementary material for: Comparative evaluation of iodinated, nanoparticle-based, and gadolinium-based contrast agents in computed tomography for small animals
Source: PLoS One. 2026 Jun 29;21(6):e0350386. doi: 10.1371/journal.pone.0350386 (PMC13313363; doi:10.1371/journal.pone.0350386)
Supplement: Attachment — Submitted filename: Response to Reviewers.docx [file pone.0350386.s009.docx]

## Response to academic editor

### Journal Requirements:

**1. Please ensure that your manuscript meets PLOS ONE's style requirements, including those for file naming. The PLOS ONE style templates can be found at** [**https://journals.plos.org/plosone/s/file?id=wjVg/PLOSOne_formatting_sample_main_body.pdf**](https://journals.plos.org/plosone/s/file?id=wjVg/PLOSOne_formatting_sample_main_body.pdf) **and** [**https://journals.plos.org/plosone/s/file?id=ba62/PLOSOne_formatting_sample_title_authors_affiliations.pdf**](https://journals.plos.org/plosone/s/file?id=ba62/PLOSOne_formatting_sample_title_authors_affiliations.pdf)

We thank the editor for noting this. We have reviewed the formatting guidelines and revised the manuscript accordingly to comply with all PLOS ONE style requirements, including file naming and structural formatting using the provided templates.

**2. To comply with PLOS One submissions requirements, in your Methods section, please provide additional information regarding the experiments involving animals and ensure you have included details on (1) methods of sacrifice, and (2) efforts to alleviate suffering.**

We appreciate this comment and have now incorporated the requested information. Specifically, we have added comprehensive details regarding (1) methods of sacrifice and (2) efforts to alleviate suffering in the "Animal Study" section of the Methods. These additions ensure full compliance with PLOS One submission requirements.

**3. Please update your submission to use the PLOS LaTeX template. The template and more information on our requirements for LaTeX submissions can be found at** [**http://journals.plos.org/plosone/s/latex**](http://journals.plos.org/plosone/s/latex)**.**

We have reformatted the entire manuscript using the official PLOS LaTeX template in accordance with the submission requirements

**4. We note that the grant information you provided in the ‘Funding Information’ and ‘Financial Disclosure’ sections do not match.**

**When you resubmit, please ensure that you provide the correct grant numbers for the awards you received for your study in the ‘Funding Information’ section.**

We have reviewed and corrected the funding information to ensure consistency between the "Funding Information" and "Financial Disclosure" sections, with all grant numbers now accurately reflected in the "Funding Information" section.

**5. Thank you for stating the following financial disclosure:**

**“This project received funding from the European Union’s Horizon 2020 research and innovation programme under grant agreements no. 670261 (ERC Advanced Grant) and 668532 (Click-It), the Lundbeck Foundation, the Novo Nordisk Foundation, the Innovation Fund Denmark, the Neuroendocrine Tumor Research Foundation, the Danish Cancer Society, Arvid Nilsson Foundation, the Neye Foundation, the Sygeforsikringen danmark, the Research Foundation of Rigshospitalet, the Danish National Research Foundation (grant 126) - PERSIMUNE, the Research Council of the Capital Region of Denmark, the Danish Health Authority, the John and Birthe Meyer Foundation and Research Council for Independent Research. Andreas Kjaer is a Lundbeck Foundation Professor. No other potential conflicts of interest relevant to this article exist.”**

**Please state what role the funders took in the study. If the funders had no role, please state: "The funders had no role in study design, data collection and analysis, decision to publish, or preparation of the manuscript." If this statement is not correct you must amend it as needed.**

**Please include this amended Role of Funder statement in your cover letter; we will change the online submission form on your behalf.**

We thank you for this clarification. We have added the following statement to the cover letter: “The funders had no role in study design, data collection and analysis, decision to publish, or preparation of the manuscript.”

**6. PLOS requires an ORCID iD for the corresponding author in Editorial Manager on papers submitted after December 6th, 2016. Please ensure that you have an ORCID iD and that it is validated in Editorial Manager. To do this, go to ‘Update my Information’ (in the upper left-hand corner of the main menu), and click on the Fetch/Validate link next to the ORCID field. This will take you to the ORCID site and allow you to create a new iD or authenticate a pre-existing iD in Editorial Manager.**

The corresponding author’s ORCID iD has been validated in Editorial Manager as required.

**7. Please include a separate caption for each figure in your manuscript.**

All figure and table captions have been separated from the figures and now appear as standalone captions immediately after the paragraph in which they are first cited. No figures are embedded in the manuscript text

**8. Please include captions for your Supporting Information files at the end of your manuscript, and update any in-text citations to match accordingly. Please see our Supporting Information guidelines for more information:** [**http://journals.plos.org/plosone/s/supporting-information**](http://journals.plos.org/plosone/s/supporting-information)**.**

Captions for all Supporting Information files have been added at the end of the manuscript, and all related in‑text citations have been updated to match the revised numbering.

**9. If the reviewer comments include a recommendation to cite specific previously published works, please review and evaluate these publications to determine whether they are relevant and should be cited. There is no requirement to cite these works unless the editor has indicated otherwise.**

The reviewers did not recommend citation of any specific previously published works; therefore, no additional citations were required.

## Response to Reviewers:

### Reviewer #1:

**The author addresses the issue of low soft tissue resolution in small animal CT imaging by proposing the use of a contrast agent to enhance the visualization of vital organs. This approach demonstrates certain practicality and innovation. Based on the results, intraperitoneal injection of the CT contrast agent can, to some extent, improve the delineation of certain organs, with a significant increase in Hounsfield Units (HU). However, the overall completeness of the study remains insufficient. The following suggestions are provided for reference:**

**1. From the perspective of animal CT imaging, organs such as the heart, lungs, and liver, being relatively large, are not particularly difficult to distinguish through non-contrast CT. In contrast, smaller organs such as the kidneys, stomach, and gallbladder pose greater challenges for differentiation. It is recommended to include data on the in vivo HU changes of these and other major organs to assess whether the contrast agent enhances their visualization.**

We have expanded the analysis to address this point. In addition to the previously reported HU values for the liver, kidney, and blood, we now include HU measurements for the stomach and spleen across the different contrast agent conditions.
These data allow for a more complete assessment of contrast‑enhanced visualization in smaller and more challenging organs. Figures have been updated to include the stomach and spleen (figures 3, 4, and 5).

**2. There is a substantial difference between intraperitoneal injection and intravenous administration. Intravenous injection is largely influenced by factors such as the distribution and retention of the contrast agent, as well as the timing of imaging. It is worth exploring whether these factors have been investigated in this study.**

We have expanded the manuscript to clarify the rationale for prioritizing intraperitoneal (IP) administration and to place it in context relative to intravenous (IV) delivery. In mice, many clinically used iodinated agents show rapid washout following IV injection, resulting in short enhancement windows and limited soft‑tissue contrast. We now explain that IP administration was selected to provide longer peritoneal residence time, outlining of abdominal organ boundaries, and a practical, cost‑efficient alternative for manual segmentation workflows.

We also added explicit details on timing depending on the protocol used. Multiple post‑injection time points were tested for administration, in terms of the five consecutive scans after injection.

**3. In clinical practice, contrast-enhanced CT is primarily used for detecting lesions. In this study, the purpose of contrast-enhanced animal CT is to achieve clearer visualization of organs. However, does this approach potentially affect the detection of lesions in practical applications? Alternatively, should a comparative experiment focusing on lesion visualization be included?**

We thank you for your observation. The study has been revised to clarify that lesion detection is outside the intended scope of this work. Our objective is to improve soft‑tissue boundary delineation to support common preclinical workflows such as tracer biodistribution analysis, organ segmentation, and dosimetry. These applications primarily require consistent visualization of organ boundaries rather than intravascular enhancement patterns.

Including a specific disease or tumor model would substantially narrow the general applicability of the method, as lesion detectability varies widely with tumor type, vascularity, necrotic areas, and other microenvironmental factors. Addressing lesion detection rigorously would require multiple disease models, which is beyond the scope of this methodological study. To maintain broad utility across laboratories, we focus on an approach that enhances anatomical visualization without requiring disease‑specific protocols.

### Reviewer #2:

**Reviewer Report: Comparative Evaluation of Contrast Agents in Small Animal CT

1. Summary
This study systematically compares the performance of clinical contrast agents, including both iodinated and gadolinium-based options, against nanobody-based preclinical agents to enhance soft tissue contrast in small animal CT. To evaluate energy-dependent contrast efficacy, the authors conducted a rigorous evaluation across phantom and in vivo mouse models using both 50 kV and 80 kV protocols. The research provides significant practical value by demonstrating a cost-effective methodology, specifically the use of diluted, affordable clinical contrast agents for intraperitoneal (IP) injection as a viable replacement for expensive preclinical alternatives. Overall, the experimental design is robust, and the inclusion of comprehensive Supporting Information successfully clarifies the impact of different energy levels (kV) on imaging outcomes.

However, to ensure the academic rigor and accuracy of the study, several critical revisions and data clarifications are required. While the authors propose a "dilution strategy," the manuscript lacks a clearly defined quantitative threshold for the "minimum effective dose" necessary for reliable automated segmentation. Furthermore, given the significant drop in contrast efficacy at higher energy levels, the discussion regarding the clinical translatability of this model must be strengthened. Most importantly, a clear discrepancy exists between the main text and the Supporting Information regarding the peak time for renal contrast enhancement; this inconsistency in the temporal data must be meticulously reviewed and corrected to maintain the credibility of the results.**

We thank the reviewer for the thorough evaluation and constructive feedback. We address all points below.

**2. Major Comments (Critical Analysis)
• Validity of the Dilution Strategy:
- While the authors advocate for dilution to reduce costs and iodine burden, the data in Fig 4 and Fig S3 indicate that beyond a certain ratio (e.g., 1:9 or 1:19), the contrast enhancement becomes nearly indistinguishable from the control.
- The logic that maintaining volume (200 $\mu$L) via dilution ensures uniform peritoneal distribution is sound, but the manuscript lacks a statistical threshold for the "minimum effective dose" required for reliable organ segmentation.**

Thank you for putting this to our attention. To address this point, we now define a minimum effective dose (MED) of 2 based on the normalized HU Mean. This threshold is defined as the minimum contrast level at which an effect is observed. This analysis has been added to the Methods and Materials; the corresponding results are presented in Figure 5 and S4 Fig.

**• Energy-Dependent Performance (50 kV vs. 80 kV):
- Analysis of Fig S1 and S3 reveals that HU values significantly drop at 80 kV compared to 50 kV due to reduced photoelectric effects at higher energies.
- Given that clinical CTs operate at much higher energies, the utility of this "diluted IP model" for generating translatable AI segmentation datasets may be limited to low-voltage preclinical settings.**

Thank you for the relevant observation. The study is not intended to translate contrast‑enhanced preclinical CT protocols to clinical imaging. We now clarify that the aim is to provide a practical contrast‑enhancement strategy specifically for preclinical systems, which typically operate at much lower tube voltages, such as 50 kV and 80 kV. Within this preclinical range, the energy‑dependent results remain directly relevant and valid for the research community.

To avoid overstating clinical relevance, we have removed the introduction and discussion of AI‑driven segmentation and now focus the manuscript on improving manual segmentation efficiency in preclinical workflows. AI is retained only for future‑use perspectives.

**• Temporal Dynamics and Wash-out:
- Time-series images (Fig 5 and Fig S4) successfully trace the contrast agent's path from the peritoneum through the kidneys to the bladder.
- Discrepancy Noted: Fig S2 (Supporting Information) shows peak renal HU values between 14–28 minutes, whereas the main text (line 204) cites 33–44 minutes. This inconsistency in "peak window" reporting must be addressed.**

We have reviewed the discrepancy and agree that the peak‑time reporting required clarification. The difference arises from the temporal resolution inherent to the two CT protocols: 7 minutes for the 80 kV and 11 minutes for the 50 kV.

Fig S2 (Supporting Information) uses the 80 kV protocol and therefore provides greater temporal sampling, and Figure 3 uses the 50 kV protocol, yielding broader effective time windows. This causes the apparent HU peaks to blur when comparing the datasets directly. To address this, the manuscript now clarifies and explains the source of this variation and reports a unified peak window of approximately 30 minutes across protocols. The main text has been updated for consistency.

**3. Minor Comments & Technical Details
• Agent Selection: Gadovist (gadolinium-based) performed poorly compared to iodinated agents and is significantly more expensive; its inclusion as a "cost-effective" candidate should be more critically dismissed in the discussion.**

We have revised the manuscript to clarify the role of Gadovist in this study. The updated text now states explicitly that Gadovist shows substantially lower soft‑tissue enhancement than the iodinated agents under the tested conditions and is not cost‑effective relative to diluted iodinated contrast. Its inclusion is now framed solely as a comparative reference point, and we no longer position it as a viable cost‑effective alternative; although we note that it remains less expensive than nanoparticle‑based preclinical agents.

**• AI Training Limitations: While IP injection is excellent for defining "organ boundaries" because the agent surrounds the organs, it is inherently unsuitable for detecting "intra-organ pathologies," a limitation that should be explicitly stated.**

We have added an explicit statement noting that IP contrast is designed to enhance organ boundary delineation and is not suitable for identifying intra‑organ lesions. Lesion‑focused AI models would require datasets generated using contrast approaches that provide intravascular enhancement rather than peritoneal outlining. However, AI training considerations have been restricted to future perspectives only.

**• Reproducibility: The Methods section provides sufficient detail regarding injection volumes (200 $\mu$L for IP, 100 $\mu$L for IV) and reconstruction algorithms to allow for independent replication.**

We appreciate the reviewer’s assessment and confirm that the methodological details remain unchanged.

**4. Formal Questions to the Authors
1) Quantitative Threshold for Dilution: In Fig 4B-D, contrast efficacy drops sharply with higher dilution. What specific quantitative metric (e.g., CNR or SNR) did you use to determine that a 1:4 or higher dilution remains "effective" for automated segmentation?**

Thank you for this helpful observation. We agree that for some organs, the effectiveness of contrast‑agent dilution was not clearly visible in the original presentation. To address this, we introduced a MED criterion based on the normalized HU signal relative to background, as described earlier.
Specifically, we now define an effective dilution as one where the normalized organ signal is at least twice the background level, ensuring a measurable and biologically relevant enhancement.

We have added a new figure (now Figure 5, with the previous Figure 5 renumbered as Figure 6). In this figure, all organ values are normalized, and the MED threshold is indicated.

**2) Biological Impact of IP Retention: Since the contrast agent remains in the peritoneal space for an extended period (as shown in Fig 5), have you considered the potential for osmotic shifts or peritoneal irritation during longitudinal studies?**

Thank you for raising this important point.

We have added clarification to the discussion addressing the potential biological impact of repeated IP dosing. And as with other administration routes, repeated dosing may necessitate switching to alternative approaches. For longitudinal imaging, we recommend using an IV‑administered nanoparticle agent rather than repeated IP injections. The nanoparticle contrast agent used in this study (Exitron 12000) is taken up by macrophages and provides stable, long‑lasting enhancement, making it well-suited for longitudinal protocols while minimizing peritoneal irritation.

**3) Discrepancy in Peak Timing: Fig S2(B) shows renal peaks at 14–28 minutes, while the text (line 204) mentions 33–44 minutes. Could you reconcile these data points and provide a standardized "optimal imaging window" recommendation?**

As mentioned above (*Temporal Dynamics and Wash-out comment),* we have re‑evaluated the datasets with respect to the different temporal resolution of the two CT protocols. We have updated the Results and Discussion to present this unified optimal imaging window at approximately 30 minutes and explained the source of the initial variation.

**4) Clinical Translatability Roadmap: While IP injection is a practical tool for small animal segmentation, how exactly do you envision these "peritoneum-defined" datasets improving diagnostic outcomes in human clinical environments where IV administration is the standard?**

The manuscript has been revised to clarify that this work is not intended to translate IP contrast protocols into clinical diagnostics. Our objective is to provide an accessible and cost‑efficient contrast‑enhancement approach specifically for preclinical environments, where IP administration is routinely used. By improving soft‑tissue boundary delineation, the method aims to facilitate faster and more reliable manual segmentation in routine preclinical workflows. This method can increase the quality of future translatable preclinical work.

### Reviewer #3:

**The manuscript is interesting, but applications of the contrast agent should be revised. In the current version, simple tests of contrast agents (Omnipaque-iohexol, Visipaque, Iomeron, Gadovist (conventionally for MRI) and Exitron nano 12000) are described. At least, the application to at least one "disease model" should be revised.

(1) Authors mentioned that there approach is 'comparative' such as IP and IV. However, this is just a choice of loading the agent. It cannot be a broad and comprehensive "comparative" approach.**

We thank the reviewer for the valuable comment. We agree and have revised the wording to avoid implying a comprehensive comparative framework. The manuscript now states clearly that we compare contrast formulations and administration routes within their practical usage contexts; specifically, IP administration for clinical iodinated agents and IV administration for nanoparticle agents.

**(2) What is the target disease? This should be clearly explained.**

Thank you for this observation. Our approach is intentionally disease‑agnostic. The goal of the study is to improve soft‑tissue boundary delineation for preclinical workflows, such as tracer biodistribution, organ segmentation, and dosimetry, that apply broadly across many disease models. A single target disease would narrow the general applicability of the method and would require validation across multiple pathological contexts to be meaningful, which is beyond the scope of this methodological study.

We now state this explicitly in the manuscript.

**(3) Authors mentioned about segmentation of CT images. However, there are no data about image segmentation.**

We thank the author for putting this to our attention. We have revised the manuscript to remove any implication that automated segmentation results are presented in this study. The diluted IP contrast method is now described exclusively as a boundary‑enhancement approach designed to facilitate future manual segmentation workflows rather than demonstrating automated segmentation performance directly. AI‑based segmentation is referenced solely as a future perspective.

**(4) Strictly speaking, Nanobody-based approach looks like iodinated approach. I think authors should distinguish (1) iodinated-approach, and (2) Gd-based approach, clearly. Or authors should suggest a different way.**

We have revised the manuscript to clearly distinguish the different classes of contrast agents evaluated in the study. Specifically, we now consistently refer to:

- Iodinated clinical agents (Omnipaque, Visipaque, Iomeron)
- Gadolinium‑based clinical agents (Gadovist)
- Nanoparticle iodine agents (ExiTron nano 12000)

All incorrect references to “nanobody‑based” agents have been removed, and terminology has been standardized throughout the manuscript to avoid confusion between these categories.

**(5) ExiTron nano 12000 is also a commercialized one, which is not novel. So, I recommend that authors should point out their novelty clearly or re-design their approach.**

We agree that ExiTron nano 12000 itself is not novel. The novelty of this work lies in the methodological framework, not in the development of a new contrast agent. We have clarified this in the manuscript. Specifically, the study contributes novelty through:

- A  comparison of clinical iodinated agents, a gadolinium‑based agent, and a nanoparticle iodine agent under matched preclinical conditions.
- A cost‑efficient diluted IP protocol that enables practical soft‑tissue enhancement using inexpensive clinical agents.
- A generalizable, disease‑agnostic workflow that improves organ boundary visibility and supports segmentation‑oriented preclinical imaging

These clarifications have been added to more explicitly state the methodological contribution and novelty of the study.

**(6) ExiTron nano 12000 is "nanoparticle"-based contrast agent, not nanobody-based agent. In the manuscript, where is "nanobody" incorporated?**

We appreciate the reviewer pointing out this error. ExiTron nano 12000 is indeed nanoparticle‑based, not nanobody‑based. The incorrect terminology has been removed, and all instances of “nanobody” have been corrected to accurately describe the agent as nanoparticle‑based.
