# Supplementary material for: Comparative evaluation of iodinated, nanoparticle-based, and gadolinium-based contrast agents in computed tomography for small animals
Source: PLoS One. 2026 Jun 29;21(6):e0350386. doi: 10.1371/journal.pone.0350386 (PMC13313363; doi:10.1371/journal.pone.0350386)
Supplement: Peer review history

# Peer review: Decision Letter 0

**DOI:** 10.1371/journal.pone.0350386.r001

**Authors:** Hesham Zakaly

24 Feb 2026

PONE-D-26-04326

Comparative Evaluation of Iodinated, Nanobody-based, and Gadolinium-based Contrast Agents in Computed Tomography for Small Animals

PLOS One

Dear Dr. Kjær,

Thank you for submitting your manuscript to PLOS ONE. After careful consideration, we feel that it has merit but does not fully meet PLOS ONE’s publication criteria as it currently stands. Therefore, we invite you to submit a revised version of the manuscript that addresses the points raised during the review process.

Please submit your revised manuscript by Apr 10 2026 11:59PM. If you will need more time than this to complete your revisions, please reply to this message or contact the journal office at plosone@plos.org. When you're ready to submit your revision, log on to https://www.editorialmanager.com/pone/ and select the 'Submissions Needing Revision' folder to locate your manuscript file.

Please include the following items when submitting your revised manuscript:

- A letter that responds to each point raised by the academic editor and reviewer(s). You should upload this letter as a separate file labeled 'Response to Reviewers'.
- A marked-up copy of your manuscript that highlights changes made to the original version. You should upload this as a separate file labeled 'Revised Manuscript with Track Changes'.
- An unmarked version of your revised paper without tracked changes. You should upload this as a separate file labeled 'Manuscript'.

If you would like to make changes to your financial disclosure, please include your updated statement in your cover letter. Guidelines for resubmitting your figure files are available below the reviewer comments at the end of this letter.

If applicable, we recommend that you deposit your laboratory protocols in protocols.io to enhance the reproducibility of your results. Protocols.io assigns your protocol its own identifier (DOI) so that it can be cited independently in the future. For instructions see: https://journals.plos.org/plosone/s/submission-guidelines#loc-laboratory-protocols. Additionally, PLOS ONE offers an option for publishing peer-reviewed Lab Protocol articles, which describe protocols hosted on protocols.io. Read more information on sharing protocols at https://plos.org/protocols?utm_medium=editorial-email&utm_source=authorletters&utm_campaign=protocols.

We look forward to receiving your revised manuscript.

Kind regards,

Hesham M.H. Zakaly, Ph.D.

Academic Editor

PLOS One

Journal Requirements:

When submitting your revision, we need you to address these additional requirements.

1. Please ensure that your manuscript meets PLOS ONE's style requirements, including those for file naming. The PLOS ONE style templates can be found at

https://journals.plos.org/plosone/s/file?id=wjVg/PLOSOne_formatting_sample_main_body.pdf and

https://journals.plos.org/plosone/s/file?id=ba62/PLOSOne_formatting_sample_title_authors_affiliations.pdf

2. To comply with PLOS One submissions requirements, in your Methods section, please provide additional information regarding the experiments involving animals and ensure you have included details on (1) methods of sacrifice,  and (2) efforts to alleviate suffering.

3. Please update your submission to use the PLOS LaTeX template. The template and more information on our requirements for LaTeX submissions can be found at http://journals.plos.org/plosone/s/latex.

4. We note that the grant information you provided in the ‘Funding Information’ and ‘Financial Disclosure’ sections do not match.

When you resubmit, please ensure that you provide the correct grant numbers for the awards you received for your study in the ‘Funding Information’ section.

5. Thank you for stating the following financial disclosure:

“This project received funding from the European Union’s Horizon 2020 research and innovation programme under grant agreements no. 670261 (ERC Advanced Grant) and 668532 (Click-It), the Lundbeck Foundation, the Novo Nordisk Foundation, the Innovation Fund Denmark, the Neuroendocrine Tumor Research Foundation, the Danish Cancer Society, Arvid Nilsson Foundation, the Neye Foundation, the Sygeforsikringen danmark, the Research Foundation of Rigshospitalet, the Danish National Research Foundation (grant 126) - PERSIMUNE, the Research Council of the Capital Region of Denmark, the Danish Health Authority, the John and Birthe Meyer Foundation and Research Council for Independent Research. Andreas Kjaer is a Lundbeck Foundation Professor. No other potential conflicts of interest relevant to this article exist.”

Please state what role the funders took in the study.  If the funders had no role, please state: "The funders had no role in study design, data collection and analysis, decision to publish, or preparation of the manuscript."

If this statement is not correct you must amend it as needed.

Please include this amended Role of Funder statement in your cover letter; we will change the online submission form on your behalf.

6. PLOS requires an ORCID iD for the corresponding author in Editorial Manager on papers submitted after December 6th, 2016. Please ensure that you have an ORCID iD and that it is validated in Editorial Manager. To do this, go to ‘Update my Information’ (in the upper left-hand corner of the main menu), and click on the Fetch/Validate link next to the ORCID field. This will take you to the ORCID site and allow you to create a new iD or authenticate a pre-existing iD in Editorial Manager.

7. Please include a separate caption for each figure in your manuscript.

8. Please include captions for your Supporting Information files at the end of your manuscript, and update any in-text citations to match accordingly. Please see our Supporting Information guidelines for more information: http://journals.plos.org/plosone/s/supporting-information.

9. If the reviewer comments include a recommendation to cite specific previously published works, please review and evaluate these publications to determine whether they are relevant and should be cited. There is no requirement to cite these works unless the editor has indicated otherwise.

[Note: HTML markup is below. Please do not edit.]

Reviewers' comments:

Reviewer's Responses to Questions

**Comments to the Author**

1. Is the manuscript technically sound, and do the data support the conclusions?

The manuscript must describe a technically sound piece of scientific research with data that supports the conclusions. Experiments must have been conducted rigorously, with appropriate controls, replication, and sample sizes. The conclusions must be drawn appropriately based on the data presented.

Reviewer #1: Yes

Reviewer #2: Yes

Reviewer #3: Yes

**********

2. Has the statistical analysis been performed appropriately and rigorously?

Reviewer #1: Yes

Reviewer #2: Yes

Reviewer #3: Yes

**********

3. Have the authors made all data underlying the findings in their manuscript fully available?

The PLOS Data policy requires authors to make all data underlying the findings described in their manuscript fully available without restriction, with rare exception (please refer to the Data Availability Statement in the manuscript PDF file). The data should be provided as part of the manuscript or its supporting information, or deposited to a public repository. For example, in addition to summary statistics, the data points behind means, medians and variance measures should be available. If there are restrictions on publicly sharing data—e.g. participant privacy or use of data from a third party—those must be specified.

Reviewer #1: Yes

Reviewer #2: Yes

Reviewer #3: Yes

**********

4. Is the manuscript presented in an intelligible fashion and written in standard English?

PLOS ONE does not copyedit accepted manuscripts, so the language in submitted articles must be clear, correct, and unambiguous. Any typographical or grammatical errors should be corrected at revision, so please note any specific errors here.

Reviewer #1: Yes

Reviewer #2: Yes

Reviewer #3: Yes

**********

5. Review Comments to the Author

Please use the space provided to explain your answers to the questions above. You may also include additional comments for the author, including concerns about dual publication, research ethics, or publication ethics. (Please upload your review as an attachment if it exceeds 20,000 characters)

Reviewer #1: The author addresses the issue of low soft tissue resolution in small animal CT imaging by proposing the use of a contrast agent to enhance the visualization of vital organs. This approach demonstrates certain practicality and innovation. Based on the results, intraperitoneal injection of the CT contrast agent can, to some extent, improve the delineation of certain organs, with a significant increase in Hounsfield Units (HU). However, the overall completeness of the study remains insufficient. The following suggestions are provided for reference:

1. From the perspective of animal CT imaging, organs such as the heart, lungs, and liver, being relatively large, are not particularly difficult to distinguish through non-contrast CT. In contrast, smaller organs such as the kidneys, stomach, and gallbladder pose greater challenges for differentiation. It is recommended to include data on the in vivo HU changes of these and other major organs to assess whether the contrast agent enhances their visualization.

2. There is a substantial difference between intraperitoneal injection and intravenous administration. Intravenous injection is largely influenced by factors such as the distribution and retention of the contrast agent, as well as the timing of imaging. It is worth exploring whether these factors have been investigated in this study.

3. In clinical practice, contrast-enhanced CT is primarily used for detecting lesions. In this study, the purpose of contrast-enhanced animal CT is to achieve clearer visualization of organs. However, does this approach potentially affect the detection of lesions in practical applications? Alternatively, should a comparative experiment focusing on lesion visualization be included?

Reviewer #2: Reviewer Report: Comparative Evaluation of Contrast Agents in Small Animal CT

1. Summary This study systematically compares the performance of clinical contrast agents, including both iodinated and gadolinium-based options, against nanobody-based preclinical agents to enhance soft tissue contrast in small animal CT. To evaluate energy-dependent contrast efficacy, the authors conducted a rigorous evaluation across phantom and in vivo mouse models using both 50 kV and 80 kV protocols. The research provides significant practical value by demonstrating a cost-effective methodology, specifically the use of diluted, affordable clinical contrast agents for intraperitoneal (IP) injection as a viable replacement for expensive preclinical alternatives. Overall, the experimental design is robust, and the inclusion of comprehensive Supporting Information successfully clarifies the impact of different energy levels (kV) on imaging outcomes.

However, to ensure the academic rigor and accuracy of the study, several critical revisions and data clarifications are required. While the authors propose a "dilution strategy," the manuscript lacks a clearly defined quantitative threshold for the "minimum effective dose" necessary for reliable automated segmentation . Furthermore, given the significant drop in contrast efficacy at higher energy levels, the discussion regarding the clinical translatability of this model must be strengthened. Most importantly, a clear discrepancy exists between the main text and the Supporting Information regarding the peak time for renal contrast enhancement; this inconsistency in the temporal data must be meticulously reviewed and corrected to maintain the credibility of the results.

2. Major Comments (Critical Analysis)

• Validity of the Dilution Strategy:

- While the authors advocate for dilution to reduce costs and iodine burden, the data in Fig 4 and Fig S3 indicate that beyond a certain ratio (e.g., 1:9 or 1:19), the contrast enhancement becomes nearly indistinguishable from the control.

- The logic that maintaining volume (200 $\mu$L) via dilution ensures uniform peritoneal distribution is sound, but the manuscript lacks a statistical threshold for the "minimum effective dose" required for reliable organ segmentation.

• Energy-Dependent Performance (50 kV vs. 80 kV):

- Analysis of Fig S1 and S3 reveals that HU values significantly drop at 80 kV compared to 50 kV due to reduced photoelectric effects at higher energies.

- Given that clinical CTs operate at much higher energies, the utility of this "diluted IP model" for generating translatable AI segmentation datasets may be limited to low-voltage preclinical settings.

• Temporal Dynamics and Wash-out:

- Time-series images (Fig 5 and Fig S4) successfully trace the contrast agent's path from the peritoneum through the kidneys to the bladder.

- Discrepancy Noted: Fig S2 (Supporting Information) shows peak renal HU values between 14–28 minutes, whereas the main text (line 204) cites 33–44 minutes. This inconsistency in "peak window" reporting must be addressed.

3. Minor Comments & Technical Details

• Agent Selection: Gadovist (gadolinium-based) performed poorly compared to iodinated agents and is significantly more expensive; its inclusion as a "cost-effective" candidate should be more critically dismissed in the discussion.

• AI Training Limitations: While IP injection is excellent for defining "organ boundaries" because the agent surrounds the organs, it is inherently unsuitable for detecting "intra-organ pathologies," a limitation that should be explicitly stated.

• Reproducibility: The Methods section provides sufficient detail regarding injection volumes (200 $\mu$L for IP, 100 $\mu$L for IV) and reconstruction algorithms to allow for independent replication.

4. Formal Questions to the Authors

1) Quantitative Threshold for Dilution: In Fig 4B-D, contrast efficacy drops sharply with higher dilution. What specific quantitative metric (e.g., CNR or SNR) did you use to determine that a 1:4 or higher dilution remains "effective" for automated segmentation?

2) Biological Impact of IP Retention: Since the contrast agent remains in the peritoneal space for an extended period (as shown in Fig 5), have you considered the potential for osmotic shifts or peritoneal irritation during longitudinal studies?

3) Discrepancy in Peak Timing: Fig S2(B) shows renal peaks at 14–28 minutes, while the text (line 204) mentions 33–44 minutes. Could you reconcile these data points and provide a standardized "optimal imaging window" recommendation?

4) Clinical Translatability Roadmap: While IP injection is a practical tool for small animal segmentation, how exactly do you envision these "peritoneum-defined" datasets improving diagnostic outcomes in human clinical environments where IV administration is the standard?

Reviewer #3: The manuscript is interesting, but applications of the contrast agent should be revised. In the current version, simple tests of contrast agents (Omnipaque-iohexol, Visipaque, Iomeron, Gadovist (conventionally for MRI) and Exitron nano 12000) are described. At least, the application to at least one "disease model" should be revised.

(1) Authors mentioned that there approach is 'comparative' such as IP and IV. However, this is just a choice of loading the agent. It cannot be a broad and comprehensive "comparative" approach.

(2) What is the target disease? This should be clearly explained.

(3) Authors mentioned about segmentation of CT images. However, there are no data about image segmentation.

(4) Strictly speaking, Nanobody-based approach looks like iodinated approach. I think authors should distinguish (1) iodinated-approach, and (2) Gd-based approach, clearly. Or authors should suggest a different way.

(5) ExiTron nano 12000 is also a commercialized one, which is not novel. So, I recommend that authors should point out their novelty clearly or re-design their approach.

(6) ExiTron nano 12000 is "nanoparticle"-based contrast agent, not nanobody-based agent. In the manuscript, where is "nanobody" incorporated?

Overall, I can't agree to the publication of this manuscript, unless any applications to disease model is provided.

**********

6. PLOS authors have the option to publish the peer review history of their article (what does this mean?). If published, this will include your full peer review and any attached files.

If you choose “no”, your identity will remain anonymous but your review may still be made public.

**Do you want your identity to be public for this peer review?**  For information about this choice, including consent withdrawal, please see our Privacy Policy.

Reviewer #1: No

Reviewer #2: No

Reviewer #3: No

**********

[NOTE: If reviewer comments were submitted as an attachment file, they will be attached to this email and accessible via the submission site. Please log into your account, locate the manuscript record, and check for the action link "View Attachments". If this link does not appear, there are no attachment files.]

To ensure your figures meet our technical requirements, please review our figure guidelines: https://journals.plos.org/plosone/s/figures

You may also use PLOS’s free figure tool, NAAS, to help you prepare publication quality figures: https://journals.plos.org/plosone/s/figures#loc-tools-for-figure-preparation.

NAAS will assess whether your figures meet our technical requirements by comparing each figure against our figure specifications.

# Author comment: Author response to Decision Letter 1

**DOI:** 10.1371/journal.pone.0350386.r002

16 Apr 2026

A detailed response to each reviewer and editor comment has been attached to this resubmission.

Attachment Submitted filename: Response to Reviewers.docx

# Peer review: Decision Letter 1

**DOI:** 10.1371/journal.pone.0350386.r003

**Authors:** Hesham Zakaly

14 May 2026

Comparative evaluation of iodinated, nanoparticle-based, and gadolinium-based contrast agents in computed tomography for small animals

PONE-D-26-04326R1

Dear Dr. Kjær,

We’re pleased to inform you that your manuscript has been judged scientifically suitable for publication and will be formally accepted for publication once it meets all outstanding technical requirements.

Within one week, you’ll receive an e-mail detailing the required amendments. When these have been addressed, you’ll receive a formal acceptance letter and your manuscript will be scheduled for publication.

An invoice will be generated when your article is formally accepted. Please note, if your institution has a publishing partnership with PLOS and your article meets the relevant criteria, all or part of your publication costs will be covered. Please make sure your user information is up-to-date by logging into Editorial Manager at Editorial Manager® and clicking the ‘Update My Information' link at the top of the page. For questions related to billing, please contact billing support.

If your institution or institutions have a press office, please notify them about your upcoming paper to help maximize its impact. If they’ll be preparing press materials, please inform our press team as soon as possible -- no later than 48 hours after receiving the formal acceptance. Your manuscript will remain under strict press embargo until 2 pm Eastern Time on the date of publication. For more information, please contact onepress@plos.org.

Kind regards,

Hesham M.H. Zakaly, Ph.D.

Academic Editor

PLOS One

Additional Editor Comments (optional):

Reviewers' comments:

Reviewer's Responses to Questions

**Comments to the Author**

1. If the authors have adequately addressed your comments raised in a previous round of review and you feel that this manuscript is now acceptable for publication, you may indicate that here to bypass the “Comments to the Author” section, enter your conflict of interest statement in the “Confidential to Editor” section, and submit your "Accept" recommendation.

Reviewer #1: All comments have been addressed

Reviewer #3: All comments have been addressed

**********

2. Is the manuscript technically sound, and do the data support the conclusions?

The manuscript must describe a technically sound piece of scientific research with data that supports the conclusions. Experiments must have been conducted rigorously, with appropriate controls, replication, and sample sizes. The conclusions must be drawn appropriately based on the data presented.

Reviewer #1: Yes

Reviewer #3: Yes

**********

3. Has the statistical analysis been performed appropriately and rigorously?

Reviewer #1: Yes

Reviewer #3: Yes

**********

4. Have the authors made all data underlying the findings in their manuscript fully available?

The PLOS Data policy requires authors to make all data underlying the findings described in their manuscript fully available without restriction, with rare exception (please refer to the Data Availability Statement in the manuscript PDF file). The data should be provided as part of the manuscript or its supporting information, or deposited to a public repository. For example, in addition to summary statistics, the data points behind means, medians and variance measures should be available. If there are restrictions on publicly sharing data—e.g. participant privacy or use of data from a third party—those must be specified.

Reviewer #1: Yes

Reviewer #3: Yes

**********

5. Is the manuscript presented in an intelligible fashion and written in standard English?

PLOS ONE does not copyedit accepted manuscripts, so the language in submitted articles must be clear, correct, and unambiguous. Any typographical or grammatical errors should be corrected at revision, so please note any specific errors here.

Reviewer #1: Yes

Reviewer #3: Yes

**********

6. Review Comments to the Author

Please use the space provided to explain your answers to the questions above. You may also include additional comments for the author, including concerns about dual publication, research ethics, or publication ethics. (Please upload your review as an attachment if it exceeds 20,000 characters)

Reviewer #1: The author has refined the paper and addressed the raised questions, thereby meeting the requirements for publication. The data are now relatively comprehensive, and publication is recommended.

Reviewer #3: The manuscript was revised well. Authors claimed that IP injection of iodinated and gadolinium-based agents enhances soft-tissue contrast by outlining abdominal organs, which is fine for publication.

**********

7. PLOS authors have the option to publish the peer review history of their article (what does this mean?). If published, this will include your full peer review and any attached files.

If you choose “no”, your identity will remain anonymous but your review may still be made public.

**Do you want your identity to be public for this peer review?**  For information about this choice, including consent withdrawal, please see our Privacy Policy.

Reviewer #1: No

Reviewer #3: No

**********

# Editor report: Acceptance letter

**DOI:** 10.1371/journal.pone.0350386.r004

**Authors:** Hesham Zakaly

PONE-D-26-04326R1

PLOS One

Dear Dr. Kjær,

I'm pleased to inform you that your manuscript has been deemed suitable for publication in PLOS One. Congratulations! Your manuscript is now being handed over to our production team.

At this stage, our production department will prepare your paper for publication. This includes ensuring the following:

* All references, tables, and figures are properly cited

* All relevant supporting information is included in the manuscript submission,

* There are no issues that prevent the paper from being properly typeset

You will receive further instructions from the production team, including instructions on how to review your proof when it is ready. Please keep in mind that we are working through a large volume of accepted articles, so please give us a few days to review your paper and let you know the next and final steps.

Lastly, if your institution or institutions have a press office, please let them know about your upcoming paper now to help maximize its impact. If they'll be preparing press materials, please inform our press team within the next 48 hours. Your manuscript will remain under strict press embargo until 2 pm Eastern Time on the date of publication. For more information, please contact onepress@plos.org.

You will receive an invoice from PLOS for your publication fee after your manuscript has reached the completed accept phase. If you receive an email requesting payment before acceptance or for any other service, this may be a phishing scheme. Learn how to identify phishing emails and protect your accounts at https://explore.plos.org/phishing.

If we can help with anything else, please email us at customercare@plos.org.

Thank you for submitting your work to PLOS ONE and supporting open access.

Kind regards,

PLOS ONE Editorial Office Staff

on behalf of

Dr. Hesham M.H. Zakaly

Academic Editor

PLOS One
